# Embryonal Rhabdomyosarcoma Presenting as Lung Metastasis in an Adult: An Uncommon Presentation

**DOI:** 10.7759/cureus.13545

**Published:** 2021-02-24

**Authors:** Rabia Taseer, Tabeer T Ahmed

**Affiliations:** 1 Histopathology, Shiekh Zaid Hospital, Lahore, PAK; 2 Histopathology, Obeid Specialized Hospital, Riyadh, SAU; 3 Internal Medicine, Combined Military Hospital (CMH) Lahore Medical College and Institute of Dentistry, Lahore, PAK

**Keywords:** rhabdomyosarcoma, embryonal, myogenin, myo d1, retroperitoneal, lung metastasis

## Abstract

Rhabdomyosarcoma (RMS) is one of the most common soft tissue sarcomas of adolescents and young adults. Histologically, rhabdomyosarcoma is classified into embryonal, alveolar, pleomorphic, and spindle cell/sclerosing rhabdomyosarcomas with further subcategorization. More than 50% of embryonal rhabdomyosarcoma occur within head and neck. The retroperitoneum and pelvis are less common sites of involvement. Embryonal rhabdomyosarcomas affect mainly, but not exclusively, children between birth and 15 years of age. Alveolar rhabdomyosarcoma tends to affect older patients. The usual metastatic sites include lung, lymph nodes, and bone marrow. We are presenting a case of a 25-year-old male patient with symptoms of breathlessness, easy fatigability, and weight loss. On chest imaging, there were multiple lung nodules. A primary diagnosis of undifferentiated malignant neoplasm was made on lung biopsy. On immunohistochemistry, the malignant cells were positive for myogenin, myoblast determination protein 1 (MyoD1), and desmin. They were negative for neuroendocrine, germ cell, epithelial, melanocytic, and lymphoid markers. Further workup showed an abdominopelvic retroperitoneal mass on abdominal computed tomography (CT) scan. The biopsy on the retroperitoneal mass showed similar morphology and immunohistochemical profile. Unfortunately, the patient's condition deteriorated rapidly in the following weeks, and he passed away.

## Introduction

Rhabdomyosarcoma (RMS) is one of the most common soft tissue sarcomas of adolescents and young adults. It accounts for an estimated 4.5% of all childhood cancers, with an incidence of six cases per one million annually [1]. Histologically, rhabdomyosarcoma is classified into embryonal, alveolar, pleomorphic, and spindle cell/sclerosing rhabdomyosarcomas with further subcategorization [2,3]. More than 50% of embryonal rhabdomyosarcomas occur within head and neck [4]. Retroperitoneum and abdomen are less common sites [5]. It has been observed that rhabdomyosarcoma can metastasize to lymph nodes, bones, and lungs. However, this is more common in cases of alveolar rhabdomyosarcoma as compared to embryonal subtype [6]. We present a case of a 25-year-old male patient who presented with lung metastasis from a retroperitoneal embryonal rhabdomyosarcoma.

## Case presentation

**Figure 1:**
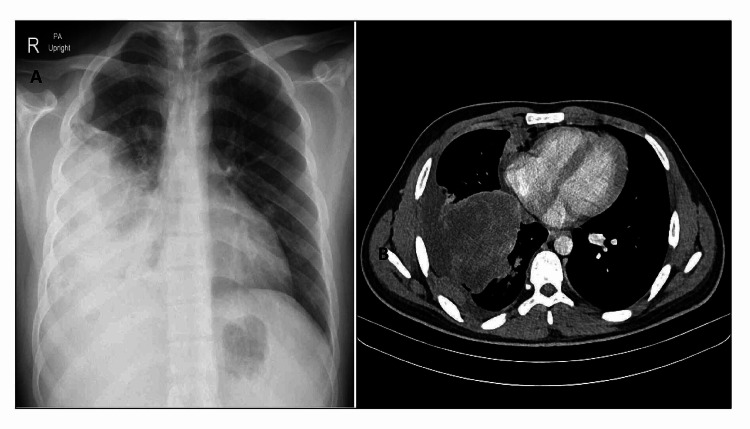
(A) Chest x-ray: right middle and lower lobe mass with right-sided pleural effusion. (B) Chest CT: right lower lobe mass with multiple smaller bilateral masses. CT, Computed tomography.

**Figure 2:**
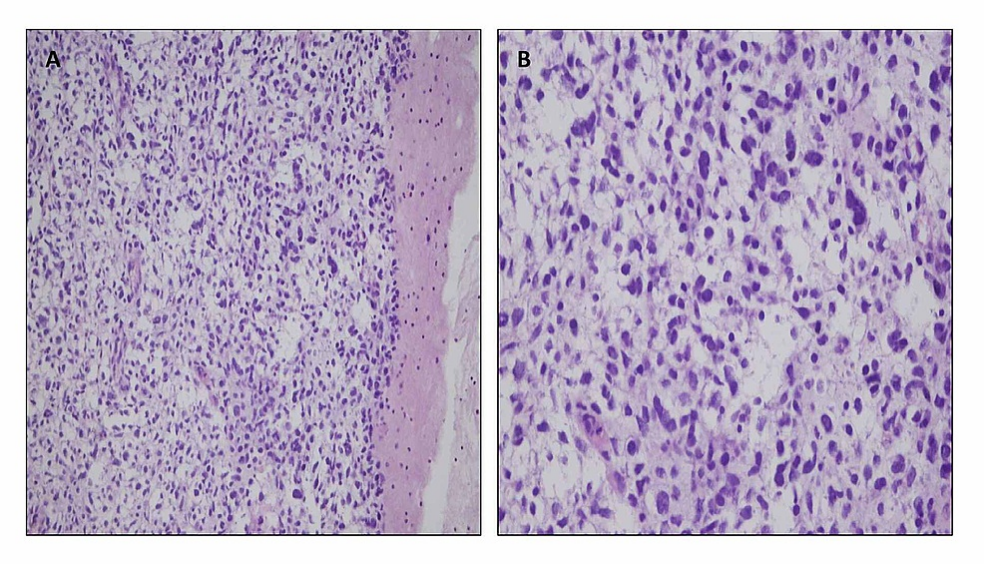
Hematoxylin and eosin (H&E) staining: cellular neoplasm showing pleomorphic cells with varying cellularity and myxoid background. (A) 20x (medium power) and (B) 40x (high power).

**Figure 3:**
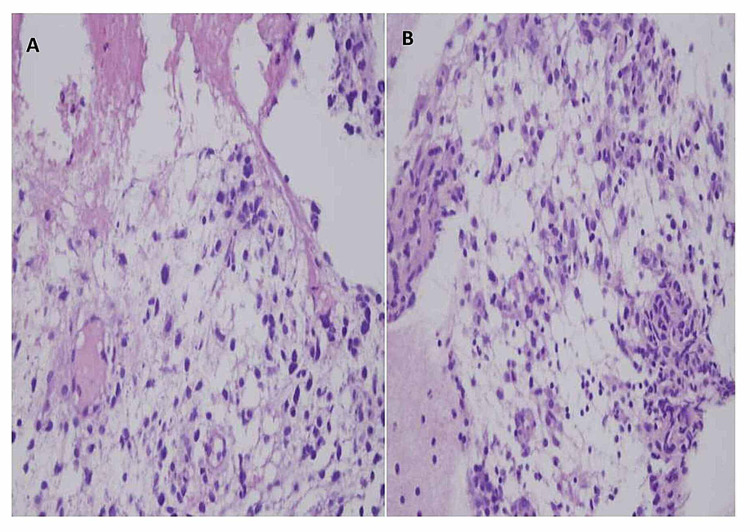
Hematoxylin and eosin (H&E) staining showing hypocellular areas and myxoid stroma. (A) 40X (high power) and (B) 20X (medium power).

A 25-year-old male patient presented to the chest clinic with easy fatigability and breathlessness for more than a month. He had a history of weight loss. The chest x-ray revealed right-sided pleural effusion (Figure 1A). On chest computed tomography (CT), there were multiple bilateral lung nodules and a 7-cm mass (with heterogeneous enhancement and non-enhancing possibly necrotic components) on the right side. There were pleural nodules (Figure 1B). A lung biopsy was performed. The histopathology department received multiple fragments of tissue in formalin; hematoxylin- and eosin-stained sections showed fragments of tissue with varying cellularity. There were densely packed, hypercellular areas (Figures 2A, 2B) and loosely textured, myxoid areas (Figures 3A, 3B). Neoplastic cells were round to oval with pleomorphic hyperchromatic nuclei. Larger atypical cells were also seen. There were no cross striations or poorly differentiated round cells.

A primary diagnosis of undifferentiated malignant neoplasm was made with a wide differential diagnosis. We considered neuroblastoma, lymphoma, and germ cell tumor. Immunohistochemistry with neuron-specific enolase, chromogranin, synaptophysin, Sal-like protein 4 (SALL4), and CD45 was done to rule out these diagnoses. Despite the unusual age bracket, the morphology was strongly suggestive of embryonal rhabdomyosarcoma, therefore myoregulatory proteins (myogenin and MyoD1) and desmin were added to the immunopanel. Cytokeratin and S100 were added to rule out the less likely differentials of carcinoma and malignant melanoma.

**Figure 4:**
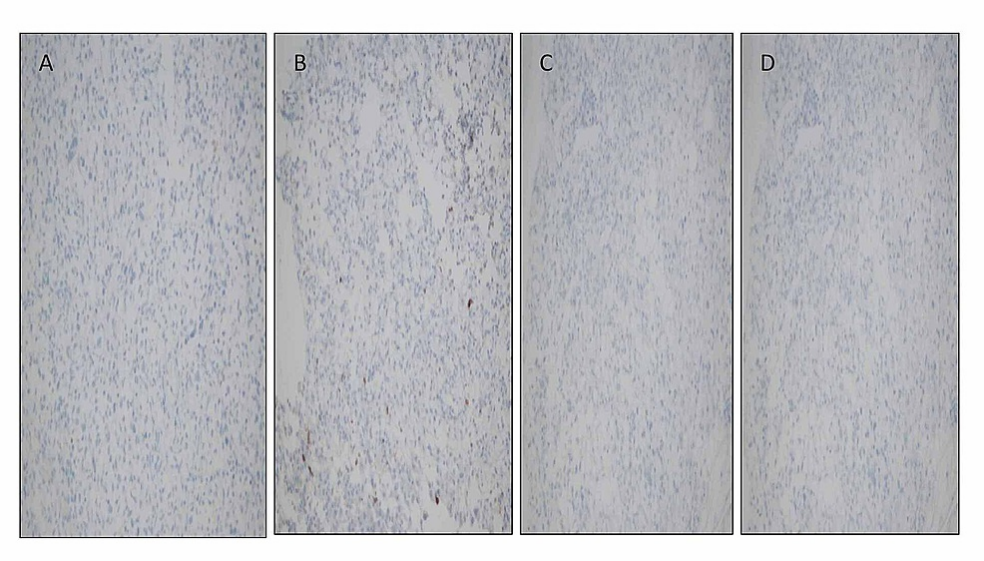
Immunohistochemical stains: (A) chromogranin, (B) S100, (C) SALL4, and (D) pan cytokeratin – negative in malignant cells. SALL4, Sal-like protein 4.

**Figure 5:**
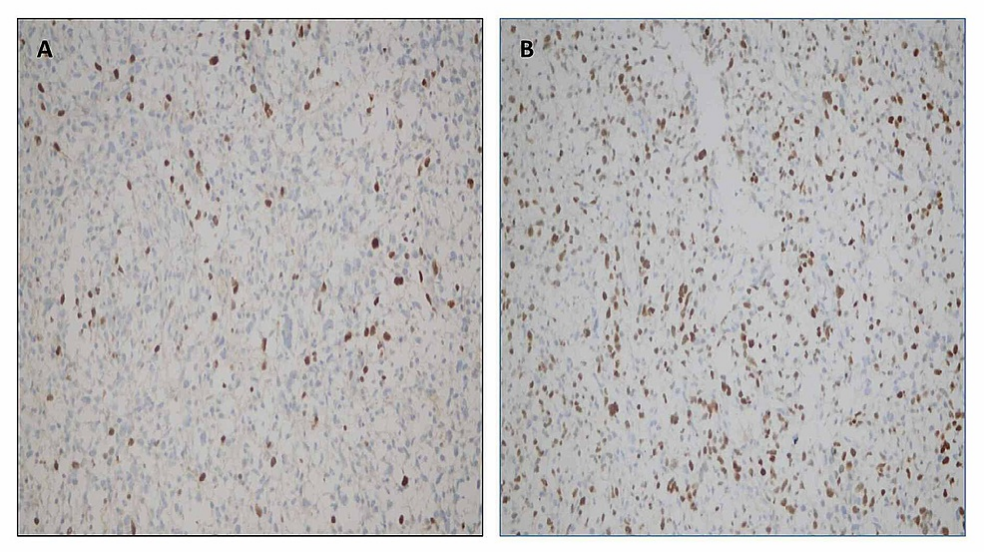
Immunohistochemistry – (A) Myogenin: positive in around 20% of tumor cells. (B) MyoD1: diffuse positivity in malignant cells.

**Figure 6:**
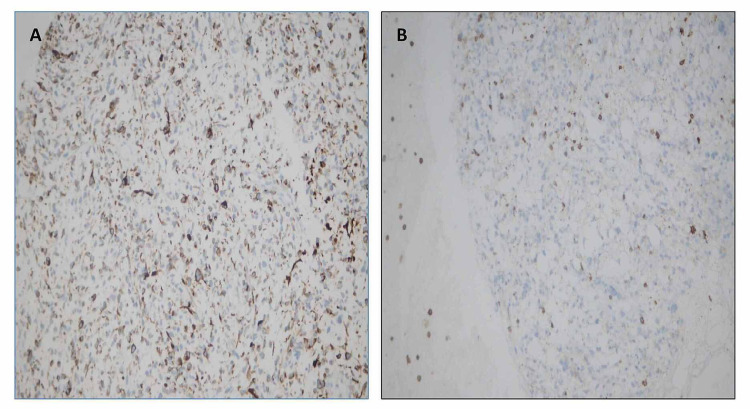
Immunohistochemistry – (A) Desmin: cytoplasmic positivity and large malignant cells. (B) CD45: negative in malignant cells.

**Figure 7:**
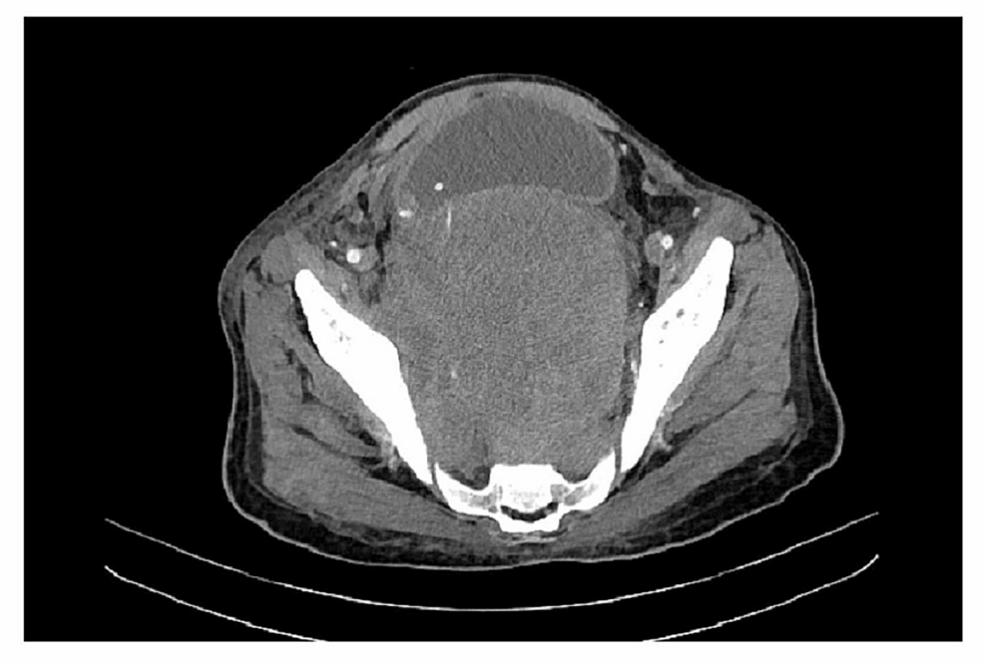
Pelvic CT scan: a retroperitoneal mass measuring 12.5 cm occupying the pelvic cavity. CT, Computed tomography.

**Figure 8:**
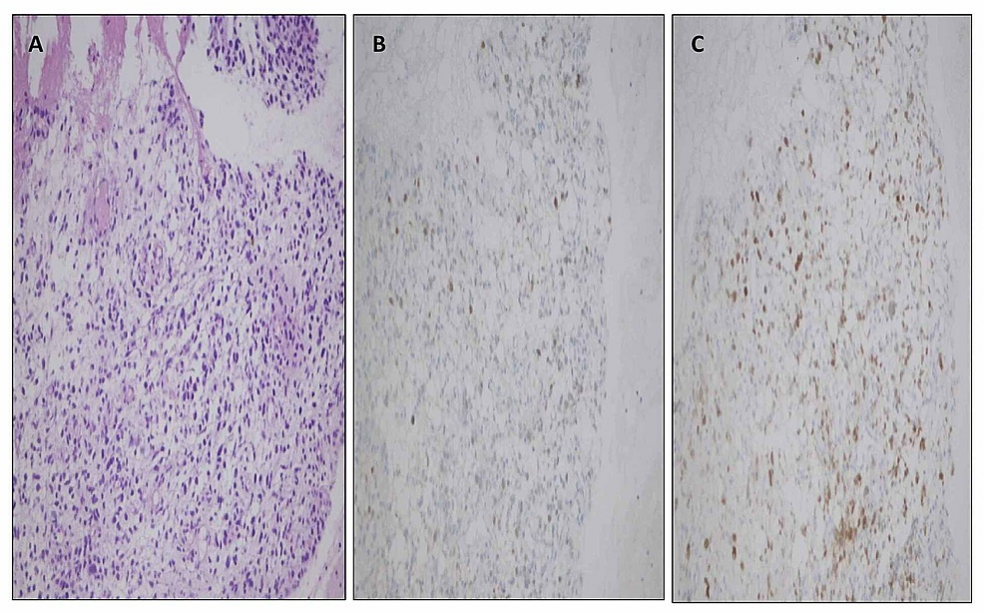
Biopsy pelvic mass – (A) hematoxylin and eosin stain, (B) myogenin immunostain, and (C) MyoD1 immunostain.

On immunohistochemistry, the tumor was negative for neuroendocrine, germ cell, epithelial, melanocytic, and lymphoid markers (Figure 4). Both myogenin and MyoD1 showed nuclear positivity in malignant cell. Myogenin expression was seen in less than 20% of tumor cells, and the intensity of staining was mild (Figure 5A). MyoD1 was more diffusely expressed (Figure 5B). Desmin showed cytoplasmic positivity (Figure 6). Based on immunohistochemistry, a diagnosis of metastatic rhabdomyosarcoma was made. The pattern of immunostain expression and morphology was considered to be consistent with embryonal rhabdomyosarcoma. After the histopathological diagnosis of embryonal rhabdomyosarcoma, the patient was evaluated by oncology department. An abdominopelvic CT was done, which showed a retroperitoneal abdominopelvic mass measuring 12.5 cm in maximum dimension, displacing rectum and urinary bladder (Figure 7). The biopsy from the retroperitoneal mass showed similar histology (Figure 8A). On immunohistocemistry the neoplastic cells showed diffuse MyoD1 positivity. They were focally positive for myogenin (Figures 8B, 8C). Desmin was positive in malignant cells. Mouse double minute 2 homolog (MDM2) and caldesmon were negative, ruling out dedifferentiated liposarcoma and leiomyosarcoma.

On follow-up, the patient's condition deteriorated rapidly within weeks following the diagnosis, and he passed away.

## Discussion

Rhabdomyosarcoma is a high-grade malignant neoplasm, with a morphologic appearance mimicking that of developing skeletal muscle. It accounts for 4.5% of all childhood cancers. The annual incidence is around six cases per one million per year [1,2]. Histologically, rhabdomyosarcoma is classified into embryonal rhabdomyosarcoma (botryoides variant and anaplastic variant), alveolar rhabdomyosarcoma (solid variant and anaplastic variant), pleomorphic rhabdomyosarcoma, and spindle cell/sclerosing rhabdomyosarcoma. Recent studies have significantly impacted this classification with the emergence of three distinct new subtypes of rhabdomyosarcomas, namely rhabdomyosarcoma with MyoD1 mutations, rhabdomyosarcoma with TFCP2 fusions, and rhabdomyosarcoma with VGLL2/NCOA2 fusions [3].

More than 50% of embryonal rhabdomyosarcomas occur within head and neck region [4]. Retroperitoneum and abdomen are less common sites [5]. In the present case the primary tumor site was retroperitoneal. Each of the rhabdomyosarcoma subtypes occurs in a characteristic age group. Embryonal rhabdomyosarcomas affects mainly children between birth and 15 years of age; it is less common in older patients. On the other hand, alveolar rhabdomyosarcoma tends to affect older patients, with peak ages of 10-25 years [6,7]. The prognosis of embryonal rhabdomyosarcoma is poorer in older age group as compared to children [8]. Our case was an older patient. A large number of patients with rhabdomyosarcoma are considered high-risk patients as they show distant metastatic disease, involving lymph nodes, bone marrow, and lungs. But metastasis has been observed more commonly in patients with alveolar as compared to embryonal rhabdomyosarcoma [9]. In our case, distant metastasis (in lung) was observed with embryonal subtype.

Morphologically, embryonal rhabdomyosarcomas are composed of primitive mesenchymal cells that show variable degrees of skeletal muscle differentiation. They are moderately cellular, but in the typical pattern they often contain both hypo- and hypercellular areas with a loose, myxoid stroma sheets of small, stellate, spindled, or round cells with scant or deeply eosinophilic cytoplasm and eccentric, small oval nuclei with a light chromatin pattern and inconspicuous nucleoli. Feature of rhabdomyoblastic differentiation (so called "strap" cells) can be seen [10]. On hematoxylin and eosin (H&E) stain, our case showed varying cellularity, alternating between densely packed, hypercellular areas and loosely textured, myxoid areas. Neoplastic cells were round to oval with pleomorphic hyperchromatic nuclei. Larger atypical cells were also seen (Figures 2, 3).There were no cross striations. No poorly differentiated round cells were seen.

Since our case was a metastatic lesion in the lung, we would not rely solely on morphology as it can be deceptive in metastatic disease. In cases of less differentiated tumors, it has been seen that the best method to detect rhabdomyoblastic differentiation of the sarcoma is the demonstration of expression of MyoD1 protein and myogenin. Dias et al. demonstrated that positive nuclear staining in both markers is an important diagnostic criterion for rhabdomyosarcoma. It is considered the gold standard in differential diagnosis of this tumor from other neoplasms. Alveolar rhabdomyosarcomas shows three times more myogenin expression than embryonal rhabdomyosarcomas. According to their data, staining for myogenin is a simple, rapid, and accurate tool for differentiating between embryonal and alveolar rhabdomyosarcomas [11]. Similarly, Rudzinski et al. and Rakhi et al. have demonstrated in separate studies that embryonal rhabdomyosarcoma shows focal myogenin immunostaining pattern, whereas myogenin expression is more diffuse in alveolar rhabdomyosarcoma. MyoD1, on the other hand, is more diffusely expressed in embryonal rhabdomyosarcoma [12,13]. Our case showed a nuclear positivity for both myoregulatory proteins (myogenin and MyoD1), but myogenin expression was seen in less than 20% of tumor cells, and the intensity of staining was mild. MyoD1 expression was more diffuse in our case (Figure 5). It is important to mention here that these antibodies also provide an alternative to molecular methods for identification of fusion-positive rhabdomyosarcomas [14,15]. Cytoplasmic expression of vimentin and desmin is expected to be positive in poor or undifferentiated cells in embryonal rhabdomyosarcomas [15]. Our case showed cytoplasmic positivity for desmin in the large pleomorphic cells. On the basis of immunohistochemistry and morphology, a diagnosis of metastatic embryonal rhabdomyosarcoma was made.

The patient was referred to the oncology department. An abdominal CT showed a retroperitoneal abdominopelvic mass. According to the literature, the most common retroperitoneal sarcoma is dedifferentiated liposarcoma followed by leiomyosarcoma (45% and 21%, respectively). Rhabdomyosarcoma is far less common in retroperitoneum [16,17]. On H&E, the morphology of retroperitoneal mass was similar to the lung mass. On immunohistochemistry, the neoplastic cells showed diffuse MyoD1 positivity. They were focally positive for myogenin and desmin. MDM2 and caldesmon were negative, ruling out dedifferentiated liposarcoma and leiomyosarcoma.

Unfortunately, the patient's condition deteriorated rapidly after the diagnosis, and he passed away two months after his presentation to the outpatient department.

## Conclusions

Embryonal rhabdomyosarcoma is considered to be more common in ages from birth to 15 years. The most common site for this malignancy is head and neck; retroperitoneum and abdomen are less common sites. Our case is an example of a retroperitoneal embryonal rhabdomysarcoma in a 25-year-old patient. The primary presentation in this case was with respiratory symptoms due to lung metastasis.
